# Radiomics: is it time to compose the puzzle?

**DOI:** 10.1007/s40336-018-0302-y

**Published:** 2018-10-15

**Authors:** Isabella Castiglioni, Maria Carla Gilardi

**Affiliations:** Institute of Molecular Bioimaging and Physiology, National Research Council (IBFM-CNR), Milan, Italy

Following the introduction of the term “Radiomics”, proposed to imply the “Comprehensive quantification of disease phenotypes by applying a large number of quantitative image features representing lesion heterogeneity and correlating with omics and clinical data” [1], the scientific literature has been flooded by a continuously increasing number of studies on this topic.

In fact, such an approach to the analysis of medical images had been adopted even before the term radiomics was coined. In some high impact studies, the potential of image features to measure lesion heterogeneity by the use of advanced image analytics (e.g., texture analysis) had been demonstrated, proving statistically significant correlation between imaging features and known “omics” prognostic factors and/or clinical endpoints, in patients with similar cancer diagnosis, e.g., [2, 3].

With radiomics, a revival of the role of in vivo medical imaging in the anti-cancer scene is seen, overlapping the Genomics era, in which Genomics seemed to be the only possible precision approach to win the cancer challenge.

Moreover, ex vivo Genomics sequencing on sample biopsies is susceptible to tissue sampling errors possibly missing detection of intra-tumor heterogeneity and consequently misleading Genomic profiles [4]. The possibility of studying cancer heterogeneity by 3D imaging of an entire lesion, in vivo, and by means of existing imaging technologies, has brought molecular imaging on the scene of personalized medicine.

Many research groups applied the radiomics approach to their retrospective oncological studies, given the immediate availability of both imaging and clinical follow-up data. Most of these exploratory studies were successful in finding a number of radiomics features correlated to clinical endopoints, in some studies allowing to stratify patients in different prognostic groups. Due to their retrospective nature, because of the lack of genetic analysis, very few studies allowed to investigate the correlation of the image traits with the Genomic profiles of the patients. The majority of studies adopted compromise solutions, such as histopathological and immunohistochemical analysis instead of Genomics.

However, this enthusiastic rush into a new research field results in a build-up of experimental and analytical work, prior to the consolidation of standardized and validated methodologies. On the one hand, many results in the literature have been obtained disregarding relevant methodological issues (a disruptive paper on this topic that is worthy of wide diffusion and reading is the one on the high risk of false discovery rate in radiomics studies [5]). On the other hand, such a scientific gym has its advantages, forcing the field to grow. A variety of results have certainly emerged, some confirmed, others contradicted, however, generally highlighting the most critical issues for a clinical translation of radiomics.

First of all, radiomics has focused attention on the need to integrate such an unprecedented amount of different skills so far and on the complexity of this cultural change. The multidisciplinary work involves the collaboration of physicians (radiologists, nuclear physicians, oncologists and other specialists), not only with biologists, physicists, and bioengineers, but, more and more, with computer scientists, biostatisticians and experts in bioinformatics. These latter expertises appear predominant in radiomics and this is perhaps the greatest novelty compared to the past. Extremely, as in [6], the images “are more than pictures: they are data” and, we can hazard, they escape the radiologists “visual inspection”. This unexpected change can perhaps be compared to what happened to biologists with the advent of Genomic analyses by high-throughput sequencing platforms. The amount of data extracted from these platforms is such that the now well-consolidated term “big data” was coined in medicine. Here, in radiomics, the number of features which can be extracted is so high, compared to conventional image analysis methods, that these new descriptors can be considered worth to enter into the “big imaging data” world.

An immediate step forward is, therefore, the translation of the feature selection procedures used so far for the analysis of big data for the analysis of radiomic data. Statistical methods are applied to avoid data redundancy, reduce the dimensionality of the problem and control false discovery rate. Procedures to guarantee the stability of the features, and consequently the generalization of radiomic results, are also set up, with respect to lesion contouring (intra/inter-operator and algorithm variability), test–retest studies (intra study variability), and independent patient cohorts.

More recently, an emerging area of investigation focusses on the dependence of radiomics results on the physical characteristics of the imaging acquisition and reconstruction systems, shifting the attention towards image protocols and processes prior to feature extraction, selection and classification, and this is the main topic for the two papers by Lovinfosse et al. in this issue [7, 8].

The instability of radiomic features on image acquisition and reconstruction protocols represents a serious constraint to multi-center radiomic studies, which have been recommended in order to achieve an adequate number of samples to guarantee the power of the radiomic results. Interestingly, a possible solution may be inspired by the Genomic analysis environment, facing the so-called “batch effect”, a variation of Genomic data caused by the experimental manipulation of the samples (different laboratories, different technicians, different measurement days). A feature standardization method has been very recently proposed based on a transformation applied to each feature separately on the basis of the batch effect of the imaging system [9].

In summary, the results that emerge consistently from the recent literature show important reductions in features due to their non-negligible variation across different conditions but the selection of a limited number of stable radiomics features is possible and leads to the definition of “Radiomics signatures” to be used as precision biomarkers for the prognosis of individual patients.

A further significant advancement in the radiomics process occurs with the application of automatic classification techniques. Intelligent systems, trained on radiomics signatures from subjects with known prognosis, allow to predict the clinical outcome for new patients, similarly to classifiers predicting prognostic gene signatures, e.g., [10]. By the definition of a radiomics profile, it might then be possible to predict the prognosis or the response to a given therapy for each individual patient, by use of automatic image analyses and in vivo. The success and enthusiasm of radiomics perhaps reach its highest level from these applications, because the impact it can have in the modern context of precision medicine is extraordinary.

However, compared to these promises, to date we recognize an insufficient level of standardization and evidence in radiomics. Even in those studies applying accurate selection criteria to produce stable radiomic profiles, significant differences exist in terms of methodology and clinical utility. There is an appreciable effort to report the results of the radiomic analysis to the “visual” control of the radiologists, for example mapping radiomics features of a particular clinical relevance at the level of the image voxel of a lesion. Thanks to these possibilities, radiologists have started interpreting the meaning of such features, on an imaging level, but we are only at the beginning of the puzzle, when one overviews all pieces and starts composing the picture using the most informative ones.

From which pieces should we start?

Statistical issues at the basis of radiomics extraction have been mostly solved, and a pipeline is now recommended to obtain radiomics profiles, e.g., [11]. Feature standardization methods have been developed and, although further confirmations are needed and their application requires specific expertise, they could be used to harmonize radiomics features from studies across different imaging systems. There is still a lack of standardization on radiomics generation (today the number of possible features of images has reached a few thousands with deep learning methods [12]) and on radiomics reporting, although an attempt of harmonization on reporting has been proposed [13]). Moreover, there is still no consensus on how to contour lesion volumes, in particular for imaging modalities with poor spatial resolution and signal-to-noise ratio (e.g., PET and DWI MRI).

Hopefully, we should now start a phase of a more systematic approach to radiomics, with the focus on “fixing the puzzle pieces in place” to guide the scientific community toward the use of a clear, shared and robust methodological framework to the radiomic process. A collaborative multidisciplinary work at international and institutional level is emerging in fact, including all the above-mentioned multidisciplinary competences (e.g., the quantitative imaging network—QIN and the image biomarker standardization initiative—IBSI [14]).

Before a collaborative multidisciplinary work is accomplished, it might be still too early to start composing the puzzle.
